# How can wellbeing at work and sustainable employability of gifted workers be enhanced? A qualitative study from a capability approach perspective

**DOI:** 10.1186/s12889-021-10413-8

**Published:** 2021-02-23

**Authors:** Patricia A.J. van Casteren, Jan Meerman, Evelien P.M. Brouwers, Arno van Dam, Jac J.L. van der Klink

**Affiliations:** 1 Tilburg School of Social and Behavioral Sciences / Tranzo, Postbus 90153, 5000 Tilburg, LE Netherlands; 2 North West University, Optentia, Vanderbijlpark, South Africa

**Keywords:** Gifted, Intelligence, Sustainable employability, Capabilities, Wellbeing, Capability approach, Qualitative

## Abstract

**Background:**

Being gifted with a very high IQ (> 98 percentile) can provide an advantage in the occupational context but can also come with its` own specific challenges. Where some studies found higher than average levels of wellbeing at work and successful careers amongst the gifted, other studies report boredom and less job satisfaction. This poses the question what gifted people value in work, and which factors are associated with the achievement of valued work related outcomes, wellbeing and sustainable employability. In this study these questions were explored using the value driven capability approach as a theoretical framework.

**Method:**

A qualitative approach was chosen and 16 in-depth semi-structured interviews with gifted workers (IQ > 130) were conducted. The transcripts were analysed using a reflexive thematic analysis aimed at identifying the work related outcomes participants aspired to achieve and the contextual and personal factors that affected the actualisation of these outcomes.

**Results:**

Participants placed great value on the opportunity to learn, to use their knowledge and skills, and tended to have high ethical standards. If realized, these values contributed to wellbeing whereas if not fulfilled, this often resulted in frustration and sadness. The most important personal factors associated with wellbeing at work and sustainable employability were the level of organizational awareness, self-knowledge, a willingness to compromise, and fear of stigmatisation. Contextually a facilitating leadership style of managers was important, allowing the worker autonomy and decision latitude. Socially, participants enjoyed others as sparring partners but often had an aversion to small talk which could lead to social avoidance and loneliness.

**Conclusions:**

If gifted workers managed (to get) what they valued in work, this was associated with wellbeing and sustainable employment Coaching aimed at improving organizational awareness, specific social skills (e.g. small talk, adaptability) and understanding their own cognitive processes could be valuable. The application of an autonomy supporting facilitative leadership style by supervisors would be beneficial. Further research should try to confirm the findings using quantitative methods and needs to examine more closely the impact of stigmatisation and leadership styles.

**Supplementary Information:**

The online version contains supplementary material available at 10.1186/s12889-021-10413-8.

## Background

Several studies in the field of highly intelligent or gifted adults agree that a high IQ provides an advantage in the occupational context, and have linked it to happiness, positive health outcomes and educational and job success [1–3]. Notable example is Terman’s classic study of the lives of gifted individuals (defined here and throughout the present study as having an IQ within the 98th percentile) in which he concluded that most of his subjects ended up as well adjusted adults and had successful careers [4, 5]. Other studies have had similar outcomes and suggest a higher than average job satisfaction for the gifted [6, 7] and label them as strong contributors to technological progress and the economic growth of organizations [8]. Also, a high IQ at the age of 13 has proven to be a good predictor of job success and satisfaction as an adult for STEM (Science, Technology, Engineering, Mathematics) oriented careers [9].

There is however also research that indicates that giftedness is no guarantee for success and wellbeing at work. Pollet and Schnell for instance differentiated between high achievers and gifted adults and found that the gifted individuals experienced significantly lower levels of wellbeing than the high achievers [10]. A Dutch survey among unemployed gifted individuals suggests that they experienced specific problems with obtaining and holding a job [11]. Other studies have shown an association with being bored at work, or found a gap between the capabilities of the worker and the requirements of the job and a proneness to workaholic behaviour [12]. All in all, being gifted appears to be a mixed blessing in the workplace that can lead to both positive and negative occupational outcomes. This poses the question which personal and contextual factors determine these different outcomes and how they affect the wellbeing at work and sustainable employability of gifted workers.

Most studies in the field appear to have focused on personal development, career choices and achievement and not as much on the topics of wellbeing and sustainable employability [13]. Yet these topics are of great importance to both the gifted workers and the organizations they work for. Employers are confronted with demographics that lead to a declining workforce [14] and have to cope with the need for flexibility and innovation. The individual worker has an obvious vested interest in staying happy and healthy at work. Hence the aim of the present study was to investigate the conditions for sustainable employability and optimal wellbeing at work of gifted workers.

Sustainable employability can be studied from various angles and theories, but recent research indicates an important role for the meaningfulness or *value* of work [15–17]. Work should ideally add value for both the worker and the organization, contribute to health and be sustainable [15]. Based upon this premise, a consortium of researchers from seven Dutch universities proposed the following definition of sustainable employability: “*Sustainable employability means that, throughout their working lives, workers can achieve tangible opportunities in the form of a set of capabilities. They also enjoy the necessary conditions that allow them to make a valuable contribution through their work, now and in the future, while safeguarding their health and welfare*.” [15]. This definition is deeply rooted in Sen’s value driven capability approach [18, 19] which stipulates that a person’s resources (e.g. health, intelligence, labour conditions) only become meaningful when he or she can convert them into outcomes he has reason to value [20, 21].. The capability set for work is the aggregation of all realistic opportunities a worker has to achieve these valued outcomes [22]. The difference between resources and what people can *actually* achieve in their (working) lives is given much attention by the capability approach. The idea that persons have different abilities to convert resources into valued outcomes is one of its core ideas [23, 24]. The factors which determine these abilities are called conversion factors and can be both personal and contextual in nature [25]. For example conversion factors like personal motivation and access to education, determine whether a resource like intelligence can be converted in a real opportunity to learn new skills.

Individual’s own capability set for work charts: (a) which work related outcomes people value, (b) whether their environment enables them to achieve these outcomes and (c) whether they are personally able to achieve them. Earlier research has shown significant correlations between the capability sets for work with individuals” sustainable employability [22, 26]..

So in order to learn more about the sustainable employability and wellbeing at work of gifted individuals, we need to look at the work related outcomes they value and the conversion factors that influence their opportunities to achieve those outcomes. This leads to the following research question: what do gifted people value in work, and which personal and contextual conversion factors are associated with the achievement of valued work related outcomes, wellbeing and sustainable employability?

## Methods

To be optimally transparent and in line with other qualitative research, the COREQ guideline was used (Consolidated criteria for reporting qualitative research: a 32 item checklist, see Additional file 1). According to this checklist, qualitative papers need to address all 32 items concerning sampling methods, setting for data collection, method of data collection, respondent validation of findings, method of recording data, description of the derivation of themes and inclusion of supporting quotations .

Since our questions were exploratory in nature, we used a qualitative research design and conducted a series of semi-structured interviews with gifted workers. This is in line with the research traditions of the capability approach, in which experiential knowledge straight from the source is valued as expertise [25]. Moreover, qualitative interviewing is a well-established method for gathering data about experiences and beliefs and its exploratory nature permits the collection of rich data [27, 28].

All interviews were conducted by two experienced interviewers, who adopted an open and non-judgmental questioning style and encouraged participants to provide examples and elaborate. All interviews started by inviting the participant to talk about what his or her present or last held job entailed. The interview was then continued in an semi-open format in which the interviewer asked questions about work related values and conversion factors. See Additional file 2 for the interview guide.

The dataset consisted of the recordings and verbatim transcripts of the interviews. Data generation and analysis occurred in parallel using a constant comparative technique with data gathered in earlier interviews, shaping the subsequent interviews [29, 30]. We conducted a reflexive thematic analysis of all transcripts using the software program Atlas.ti 7.0 and applied the phases as described by Braun and Clarke [31–33]. This process is illustrated in Table 1. The transcripts were independently and open coded by two researchers per transcript, allowing codes to emerge from the text so no information would be lost. Their coding was then compared and discussed by all five researchers until consensus was reached.

**Table 1:** Phases of the reflexive thematic analysis [33]

Phase	Description of the process
1.familiarising with data	Transcription of interviews, exporting to Atlas Ti, read en re-read
2.generating initial codes	Two researchers independently coded all transcripts. Capability set for work was used to organize data. Codes were discussed and re-checked.
3.searching for themes	Codes were collated by theme and discussed with all authors until consensus was reached.
4.reviewing themes	Check to see if codes and themes match up. Data were checked for overlap.
5.defining and naming themes	Naming of the themes and sub-themes. Check: do the themes adequately tell the overall story of the data?
6.producing the report	Final analysis, selection of compelling quotes, link to research question.

As previous research on the capability approach showed that there were seven aspects that Dutch workers valued highly in work [15, 22], this set of seven values was integrated in the topic list and was used as a coding framework for the study. The seven work related values are: The opportunity to use your knowledge and skills, to develop your knowledge and skills, to earn a good income, to be involved in important decisions, to have and build meaningful relationships, to set your own goals and to be part of the creation of something valuable [22].)

### Participants: recruitment and selection

In research, intellectual giftedness is commonly defined as having an IQ score within the 98th percentile, and this definition was also used in the present study [34] [5, 35].

A request to participate in this study was sent by email to all members of Mensa in the.

Netherlands. Mensa is an international society that requires proof of an IQ score in the 98th.

percentile of an approved intelligence test to become a member [36, 37]. Only individuals aged 18 and older were eligible. Eighty-two people expressed an interest in joining the study. In order to collect a broad spectrum of experiences, we made sure to include an equal number of men and women,

both salaried and self-employed workers and participants who were unemployed at the time of the.

interview. The prospective participants were contacted by one of the researchers who checked eligibility and set up an appointment. In total, 16 participants were interviewed. The interviews were conducted face-to-face on the university campus or at another place convenient to the participants. In order to achieve saturation, participants were recruited until saturation was reached, i.e. the new data started to replicate previously gathered data [38, 39]. All participants were informed about the purpose of the interviews before an appointment was scheduled and all signed informed consent forms. Ethical approval was obtained from the Ethical Review Board for the Social and Behavioral Sciences of Tilburg University.

#### Characteristics of the participants

A total of 16 gifted adults, 8 men and 8 women, were included in the study. The mean age of participants was 46 [standard deviation (SD) 9.59], at the time of the interviews. The youngest participant was 27, the oldest was 58. Two participants were unemployed at the time of the interview. All other participants had fulltime jobs of at least 32 h per week. Most of them held a position that matched their educational level and some had been in the same job for a long time.

Most participants had graduated with a bachelor’s or master’s degree. One participant held a.

high school diploma and one participant did not have any official diplomas which he attributed.

to not fitting into the educational system.

Characteristics of the participants’ are presented in Table 2.

**Table 2:** Characteristics of participants

Participant	Age group	F/M	Self -employed	Sector	Occupation	Currently unemployed
A	30–40	M		Logistics	Manager	
B	40–50	M	X	Proces industry	Interim Manager	
C	30–40	M		IT	IT consultant	X
D	50–60	M	X	Insurance	Medical consultant	
E	50–60	M	X	Consultancy	Consultant	
F	30–40	M		Construction	Architect	X
G	50–60	F	X	Healthcare	Massage therapist	
H	20–30	F		Government	Manager	
I	50–60	F		Education	Teacher	
J	40–50	F		IT	Project manager	
K	30–40	F		IT	It analyst	
L	40–50	F		Government	Policy adviser	
M	50–60	F	X	Healthcare	Psychologist	
N	40–50	M		Transport	Bus driver	
O	50–60	M	X	Consultancy	Accountant	
P	40–50	F	X	Media	Subtitler	

## Results

All participants welcomed the opportunity to speak confidentially about their experiences as gifted workers and were eager to discuss the topic. Most were hesitant to be open about their high intelligence in their work environments, some expressed a fear to disclose their giftedness and anticipated negative social consequences if that would happen.

You cannot put it in your cv because it will be frowned upon. (Participant E, Consultant)

You get these looks: are you really that smart? That’s an unpleasant moment, what do you tell them?(…) you have to apply some nuances and I can imagine there will be employers who feel threatened. (Participant C, IT consultant)

### Capabilities and conversion factors

#### The opportunity to use knowledge and skills

All participants indicated that they considered the opportunity *to use knowledge and skills* to be important and a vital component of job satisfaction. Participants expressed a preference for working on complex issues and using their analytical skills.

When I’m really immersed in an issue, how do all the elements fit together? How am I going to connect all of them? That’s when I’m at my best (participant D, medical consultant)

When their jobs were not challenging enough, they tried to find a challenge outside of the workplace for example by subscribing to online courses or pursuing other interests like music or art.

I’m an educated professional but I’m also really into astrology, serious astrology (….) It’s fascinating, a different way of thinking, like a matrix of time and place. (Participant I, teacher)

I’m enrolled in (academic level) online classes so at least I will be challenged in the evenings. I miss that during the day and this gives me balance. If I don’t do this, I will become frustrated, feel like I didn’t make enough of the day, it’s all too easy. (participant A, logistics manager)

This strategy did not work for all participants and a lack of opportunities to use their knowledge and skills, could then lead to sadness and frustration.

I was bubbling over with ideas and plans, which quickly lead to altercations. But I could not leave the unit. (…) it became really stifling and at times I walked down the hall crying my eyes out. (Participant L, policy advisor)

Another risk attached to this issue, was that by applying all their knowledge and skills, they often exceeded the performance levels their clients or supervisors expected.

I’m not sure it’s always a positive, a client might not want me to calculate everything up to three decimal points. Or at least he does not want me to charge him for it. *(*Participant O, accountant)

An important conversion factor for this capability was organizational awareness. Understanding the formal and informal social hierarchy and the processes within the organization, contributed to the successful achievement of this value:

They say: You are way too junior in age and experience to participate in these meetings. And I’m aware of that, but I still want to participate and contribute on a more complex level. (participant A, logistics manager)

#### The opportunity to develop knowledge and skills

Participants expressed a strong drive to learn and to experience new things. This drive to develop new skills had sometimes led to the successful completion of several academic degrees and work related courses. Several participants indicated that the opportunity to learn new things was an essential capability for them.

It’s like an insatiable hunger for new knowledge, anything new, new environments, new experiences (…) it’s like a monster living inside of you and you just have to accept it’s there. And give it some space. (Participant A, manager).

When the need to learn could not be met at work, people tended to experience frustration and boredom. Some looked for an opportunity to develop new skills outside of the work place.

And then you get bored and from boredom frustration emerges. (…) The risk is that when you don’t find that challenge, you become apathetic because of the situation, and cynical. (Participant A, manager).

Understanding their own need to learn was an important conversion factor, as well as self-confidence. An occupational environment that encourages learning and support from managers and colleagues also helped.

Now that I know that I’m gifted, the sky is the limit! It may sound a bit arrogant, but I love knowing that I will not easily reach my limit, that I can continue to learn new things. (Participant H, manager)

#### The opportunity to be involved in important decisions about your work

Most participants indicated that the opportunity to influence important work decisions was of no great importance to them. They generally preferred to focus on the content of their jobs and were happy to leave decision making up to their managers.

I would rather advise than have to weigh the pros and cons and participate in decision making. (Participant K, IT analyst).

Most participants who were not interested in influencing decisions, had managed to delegate this responsibility to colleagues or supervisors. Those who did want to participate in decision making, had often encountered resistance in organizations and several had decided to start their own businesses because of it. Organizational awareness, strong coping skills, and knowing how to choose your battles, were important conversion factors here.

I could get angry about things that aren’t going the way I would like them to go, things that are wrong, that shouldn’t happen, but I cannot affect them. I can point them out, but that’s it. And I’m okay with that. (participant N, bus driver).

You can come up with ideas but you never know if they will be executed.(…) if you want to know where my frustrations came from, that’s part of it. (participant E, consultant)

#### The opportunity to develop and maintain meaningful work relations

Participants enjoyed social interactions at work and especially appreciated having sparring partners to discuss work related issues with and to have in-depth discussions with. At the same time, most participants expressed an aversion to small talk and some actively avoided social occasions where they would be expected to engage in trivial conversations.

I like to be able to interact with my colleagues about work. I am but one person, have but one opinion, one idea and I think it’s useful when someone else can reflect upon it as well. (participant H, manager).

I’m not such a nerd or so rude that I’m not interested in your weekend plans or in how your mother is doing after another hospital stay (…) I want to know all that, but it should be concise, it doesn’t have to take 2 hours. (participant O, accountant).

Loneliness and isolation were mentioned by several participants as negative outcomes of not being able to develop and maintain meaningful relationships at work. Most had experienced problems connecting with their co-workers at some time in their careers. Awareness of the impact their high intelligence has on social interactions was mentioned as an important conversion factor, as well as a willingness to bridge the gap with co-workers by improving adaptability.

Because you are fast and the others are not (…) you have to learn that apparently your speed is not average. (participant M, psychologist).

I think it’s a big part of being gifted, you have to do a lot of thinking for other people. What do they mean? How can I explain my idea so they will understand? … So all day long you think about how other people think and you try to react to that. (participant C, IT consultant).

A judgement free work environment that does not negatively stigmatize outliers and allowed workers to be authentic, was described as another positive conversion factor.

Here I am surrounded by smart people, I can be myself, my manager supports me, not everybody has that luxury (…)I know what it’s like to work in an unwelcoming work environment where managers do not support you and co-workers gossip, it makes me feel sad, it hurts. (participant J, project manager).

#### The opportunity to set their own goals

When discussing the capability to set their own goals, participants considered the opportunity to determine how to proceed towards a goal as more important than actually setting the goal.

When someone assigns me a task, I like to determine how we could best proceed towards our goal. So I can determine the best way to get started by myself. (participant K, It analyst).

The attitude of supervisors was mentioned as an important conversion factor for this capability and participants expressed a preference for managers who adopted a facilitative management style. Managers who interfered too much with the execution of the work were described as a burden:

I just did my job and regarded my managers as facilitators (…). But they had a tendency to interfere when everything was running smoothly and that was more a burden to me than helpful. (Participant D, medical consultant)

#### The opportunity to earn a good income

Participants frequently indicated that they appreciated a steady income to cover their basic needs, but also that their career ambitions were much more focused on opportunities to learn or to work with interesting people, than on status or monetary rewards. Self-employed participants reported embarrassment when they had to send out bills, especially when they had enjoyed working on an assignment or felt like they hadn’t had to put in a lot of effort:

I think that’s how I make my choices, when it comes to jobs, I don’t always look for the best pay check, but for the best challenge. (participant A, manager).

Especially for the business owners, the insignificance of money could lead to financial difficulties. Most participants had never really experienced financial difficulties and had always managed to make a decent living. Some had to stay in a job in order to support their families and experienced this as a burden.

Money never comes first for me (…) it’s hard to send a bill for something that just pops up spontaneously. (participant E, consultant).

#### The opportunity to contribute to something valuable

Contributing to something valuable like the environment, technological progress or the wellbeing of others was very important to the participants. They expressed a strong desire to use their talents in ways that mattered and to really contribute. Participants working in a context that did not allow the achievement of this value, would often feel frustrated and look for opportunities to do so outside of work or even change jobs.

I want it to make sense, to have meaning, to be important. (participant F, architect).

I know my contributions here matter, people tell me so and that’s nice. I think it’s important that it’s always about people here and not just about processes and products.” (participant J, project manager)

I could say it’s really important what I do, but if I’m honest, well , I think it’s just busy work (…) I really feel like I have to make a change to something more meaningful. (participant K, IT analyst).

The ethical and moral standards of the organizations they worked for were considered to be very important and had to match the participants` standards. The unwillingness to compromise on ethical issues had lead to conflict and job loss for some:

You know that in construction certain [unethical] things happen, but I was really glad that my employer did not engage in those things. That’s why I could work there. (participant F, architect)

I would rather decline a profitable business opportunity than violate my principles. (participant E, consultant).

## Discussion

The aim of the present study was to explore what gifted people value in work, and which personal and contextual conversion factors are associated with the achievement of valued work related outcomes,wellbeing and sustainable employability. Participants placed great value on the opportunity to learn, to be able to use their knowledge and skills, and tended to have very high ethical standards. If they were able and enabled to work in an environment where their valued were realized, this greatly contributed to their wellbeing. Alternatively, not being able or enabled often resulted in sadness and frustration. If our participants could not achieve their capabilities at work, some managed to achieve them elsewhere, thus compensating for their unsatisfactory occupational context. We found that most participants had successfully graduated and had a job that matched their interests and IQ. Yet we also found that some of them had not been able to achieve a college level education and that several participants had experienced problems at work and unemployment or appeared to be stuck in a job that did not fit their abilities and interests. When.

Furthermore, we found that the participants valued the opportunity to develop and maintain meaningful relationships at work, but their interpretation of meaningful relationships was quite specific. They had a strong preference for sparring partners and in depth discussions while small talk was often described as a burden and some participants actively avoided informal social gatherings. This avoidance might contribute to the difficulties some of them experienced connecting with co-workers and their perceived lack of relatedness. This is a cause for concern since perceived isolation is considered a major risk factor for morbidity and mortality [40], but also because social skills are increasingly important in the labour market [41]. Work relationships also provide a buffer for stress and adversities and enable personal growth, friendship and care for each other [42, 43]. We found that self-knowledge and understanding the impact of their high IQs on social interactions, was an important conversion factor especially when participants were willing and able to adapt to their environment.

Another way in which the participants appeared to share very specific experiences, was the strength of their ethical and moral convictions. The ethical standards of the gifted have been described before [44] and the present study confirms that the opportunity to adhere to their ethical norms is also of great importance in the occupational context. Organizational awareness and the willingness to compromise and adapt, seemed to determine whether conflicts would actually arise. The willingness to compromise was not only an important conversion factor in situations that involved ethics, but also in instances where participants had suggested improvements or innovations and the drive to convey an innovative idea was considered more important than hierarchy or protocols. Several participants described their strong ethical convictions as likely causes for conflict and job loss. Compromising on their ethical and moral convictions felt like acting at the expense of their authenticity for some participants. Feeling authentic at work is strongly influenced by the degree to which employees evaluate their work environment as being similar to their own values [45]. The opportunity to be authentic at work is an important factor contributing to work related health outcomes [46].

What unexpectedly stood out was that many participants anticipated negative social consequences if they would disclose to be highly intelligent or gifted. Some went to great lengths to conceal their intelligence at work, which could be a way to avoid being stigmatized [47]. Research has shown that concealability of a stigmatized identity at work increases distress [48–50]. Supervisors, HR officers and counsellors should be aware of this phenomenon and support workers that experience problems as a result of anticipated stigma.

Our findings regarding the need for autonomy of the gifted, are in line with research in the general working population that shows that high levels of perceived control and decision latitude at work are generally associated with job satisfaction and health [51–53]. Autonomy can also be a mitigating factor for the negative effects of being overqualified for one’s job [54]. These findings from previous research coincide with the experiences of the participants in this study, but their need for control and autonomy was especially noticeable with regard to the method of working. They strongly preferred a manager who would not interfere with the process, but would arrange the prerequisite conditions for them. Amongst the general working population, a facilitative leadership style has been found to have a positive effect on team commitment and effectiveness [55] and on employee stress and affective wellbeing [56]. The present study suggests that this leadership style would be a very good fit for the gifted worker as well. Some participants who had run into conflicts at work because of their need for autonomy, had started their own businesses. Previous research has shown that entrepreneurship is often a very positive experience for the intellectually gifted [57].

### Strengths and limitations

This study has several limitations: First, we used a narrow definition of giftedness with the achieved IQ score as the only criterion. In literature there are several definitions of giftedness that acknowledge characteristics like creativity, sensitivity and task commitment besides a high IQ [58, 59]. The main goal of this study however was to explore the achievement of work values by gifted workers. We chose to use the narrow IQ-based definition of giftedness to make sure the included participants met the one criterion all definitions have in common and is as objective as possible.

A second important limitation is that participants’ IQ was not directly measured for this study, but an indirect measure was used (membership of Mensa, which is only accessible for gifted people). Generally, testing intelligence takes about 3 h and is costly, which forms practical barriers for research. As Mensa has strict and official regulations for admission [28] recruiting their members provided a good alternative. In the Netherlands, there are three ways to become a Mensa member: [1] To take a test under the supervision of one of the psychologists that is approved of by Mensa; (b) By formal attestation from a registered and qualified psychologist, proving that the candidate’s intelligence is within the 98th percentile; [2] by showing proof of previous membership of Mensa (international). Future research should collect data on the intelligence test administered and on the exact IQ, as that information was lacking in our study and may be important. A further limitation is that the study population consisted of members of Mensa, who volunteered their time. This may have comprised the generalizability of the study, as it may have led to selection bias because all participants were aware of their high intelligence and were interested in the topic. However, the aim of qualitative research is not generalizability, but rather to provide in-depth insight into a complex topic [28, 60]. Future studies should try to include gifted individuals who are not members of Mensa for example by administering an IQ test to a group of volunteers or by using data acquired by assessment centres to select participants.

Notwithstanding these limitations, this exploratory study offers a valuable insight into the experiences of the gifted at work and has thrown up interesting questions that merit further investigation.

### Implications for further research

Future research should aim to confirm the findings of the present study in a quantitative design. A research design within which a comparison could be made with people with IQs lower than 130 could also provide interesting findings. Further studies regarding the role of anticipated stigma as experienced by the highly intelligent would help with the development of strategies to cope with this issue. Further research into preferred leadership styles and decision latitude would also be valuable in order to shape the best occupational context for gifted workers. Coaching and counselling protocols specifically tailored for the coping skills the highly intelligent need at work would also be very helpful. Finally, further research on the applicability of the capability approach in the field of work and health and sustainable employability, would be helpful for designing future studies in this field.

## Conclusions

Using the capability approach as a framework, we gained valuable insight in why a high IQ may lead to job success as well as to problems at work. The participants shared some specific experiences and characteristics like a specific interpretation of social relationships, aversion to compromise regarding ethics, having to conceal their high IQs at work and a strong need for learning, using their knowledge, autonomy and decision latitude. The most important personal conversion factors that helped them achieve their valued outcomes in spite of these obstacles, were organizational awareness, self- confidence, understanding their own intelligence and the willingness to compromise. The most important contextual conversion factors were support from co-workers and a facilitative leadership style of managers. Being aware of these conversion factors, could help gifted workers prevent work related problems and allow them to thrive at work. Also professionals working with this group (HR, management, occupational health, career counsellors) should be encouraged to incorporate these insights in their interactions with the gifted in order to help them achieve valuable outcomes for themselves and for their organizations.

## Supplementary Information

**Additional file 1.**

**Additional file 2.**

## Data Availability

Due to privacy considerations, the collected data (interview recordings and transcript) are not freely available. Access to the data can be granted by the corresponding author upon reasonable request.

## Abbreviations

CA: Capability approach
