# Supplementary material for: How can wellbeing at work and sustainable employability of gifted workers be enhanced? A qualitative study from a capability approach perspective
Source: BMC Public Health. 2021 Feb 23;21:392. doi: 10.1186/s12889-021-10413-8 (PMC7901097; doi:10.1186/s12889-021-10413-8)
Supplement: Supplementary file 1 — Additional file 1. [file 12889_2021_10413_MOESM1_ESM.docx]

Domain 1: Research team and reflexivity

Personal Characteristics

1. Interviewer/facilitator

All interviews were conducted by Patricia van Casteren (PvC) and Jan Meerman (JM)

2. Credentials

PvC: MSc, licensed psychologist specializing in work and health and PhD student.

JM: Bsc (psychology and medicine), in the final stages of finishing medical school

3. Occupation

PvC: Occupational psychologist and PhD student

JM: medical student

4. Gender

PvC: female

JM: male

5. Experience and training

PvC: extensive training as a psychologist, several advanced courses, 15 years experience as a full time psychologist

JM: extensive training in medical school and psychology (BSc), experienced interviewer in research projects

Relationship with participants

6. Relationship established

All participants have received a brief outline of the study by mail and received a phone call to further explain the study and schedule an appointment. There were no prior professional relationships between interviewers and participants. (Method section, Participants: Recruitment and selection, page 7)

7. Participant knowledge of the interviewer

Participants knew about the nature of the study, the goals of the interviewer and that the interviews would be recorded and transcribed (audio only).

8. Interviewer characteristics

Participants were informed about the level of training and occupation of both interviewers

Domain 2: study design

Theoretical framework

9. Methodological orientation and Theory

Reflexive thematic analyses with the capability approach as a theoretical framework.

Participant selection

10. Sampling

Five women and five men were randomly selected from the group of people who had expressed an interest in participating. We made sure to include both salaried workers and self employed individuals. Additional participants were also selected based upon age in order to achieve a good balance within the group. (Method section, Participants: Recruitment and selection***,*** page 7)

11. Method of approach

A request to participate in this study was sent by email to all members of Mensa in the Netherlands. Mensa is an international society that requires proof of an IQ score in the 98^th^ percentile of an approved intelligence test to become a member [25]. 82 people expressed an initial interest in joining the study. All selected participants were approached by telephone to provide additional information and schedule an appointment. (method section, Participants: Recruitment and selection***,*** page 7)

12. Sample size

16 (Method section, characteristics of the participants, page 8)

13. Non-participation

nobody dropped out

14. Setting of data collection

Either at the university campus, at the workplace of the participant or at their home, whatever they preferred.

15. Presence of non-participants Was anyone else present besides the participants and researchers?

No

16. Description of sample What are the important characteristics of the sample? e.g. demographic data, date

Individuals were eligible to participate if they were at least 18 years old and had achieved a score within the 98^th^ percentile of a properly administered intelligence test.

We included eight men and eight women in the study. The mean age of participants was 46 [standard deviation (SD) 9.59], at the time of the interviews. The youngest participant was 27, the oldest was 58. Two participants were unemployed at the time of the interview. All other participants had fulltime jobs of at least 32 hours per week. (method section, characteristics of the participants***,*** page 8)

Data collection

17. Interview guide

All interviews started with the invitation to the participant to provide some information about their day-to-day work activities. Participants were encouraged to provide examples and share experiences. Interviewers used the values from the capability set for work as an interview guide to provide some structure to the interviews but the story of the participants was always paramount. This approach was pilot tested on a volunteer.

18. Repeat interviews

no

19. Audio/visual recording

Audio recording

20. Field notes

Yes, observations made during the interviews.

21. Duration

45-90 minutes, 60 minutes on average

22. Data saturation

Yes . (Method section, Participants: Recruitment and selection***,*** page 7)

23. Transcripts returned

In one case upon request of the participant

Domain 3: analysis and findings

Data analysis

24. Number of data coders How many data coders coded the data?

2

25. Description of the coding tree

Coding tree :

In order to organize and structure the themes that had emerged, the codes were labelled in accordance with the seven values from the capability set for work or as personal and contextual conversion factors. (method section, page 7)

26. Derivation of themes

Derived from the data. Capability set for work was used to structure the results after coding.

27. Software

Atlas TI (method section, page 6- 7)

28. Participant checking

No

Reporting

29. Quotations presented

yes

30. Data and findings consistent

Yes

31. Clarity of major themes

Yes

32. Clarity of minor themes

Yes
