# Supplementary material for: How can wellbeing at work and sustainable employability of gifted workers be enhanced? A qualitative study from a capability approach perspective
Source: BMC Public Health. 2021 Feb 23;21:392. doi: 10.1186/s12889-021-10413-8 (PMC7901097; doi:10.1186/s12889-021-10413-8)
Supplement: Supplementary file 2 — Additional file 2. [file 12889_2021_10413_MOESM2_ESM.docx]

**How can wellbeing at work and sustainable employability of gifted workers be enhanced? A qualitative study from a Capability approach perspective.**

**Interview guide**

Interviews are to be conducted on the University campus or at an other place convenient to the participant. Make sure there is no one in the room except for the interviewers and the participant.

- Welcome participant, introduce yourselves, offer coffee, tea or water.
- Inform participant about the nature of the study and the audio recording (this has already been explained in the telephone conversation).
- Ask participant to sign informed consent form
- Answer any questions they may have.

**Start recording**

- Help participant feel at ease by emphasizing there are no wrong answers and express genuine interest in their experiences.
- Start of by asking participant to describe a typical working day. This helps them adjust to the situation and provides some insights into the day-to-day work life.
- Always encourage participants to elaborate and ask for examples

**Ask participant about the values he wants to achieve at work.**

- Keep the seven work capabilities in the back of your mind but don`t prompt the participant.

**Ask whether his work environment facilitates the achievement of these values**,

- which obstacles does he experience and what is helpful?

**Ask whether he or she is actually able to achieve the important values?**

- Ask supplementary questions: which personal factors are helpful, which obstacles do they experience?

**Are there any specific aspects of being highly intelligent they find helpful or inconvenient?**

- If the conversation stalls, you can ask for the importance of one the seven work capabilities to get the conversation going again.
  - Capability set for work
  - The opportunity to use your knowledge and skills
  - The opportunity to develop your knowledge and skills
  - The opportunity to be involved in decisions about your work
  - The opportunity to have or build working relationships
  - The opportunity to set your own goals
  - The opportunity to earn a good income
  - The opportunity to contribute to the creation of something valuable
- Participants can stop the interview at any time.
- If participants become emotional, be supportive and pause if necessary.

**Finish the interview when time is up (60-90 minutes).**

- Thank participant for his cooperation. Advise them that the analyses is time consuming, so it may take several months before they can be informed about the results.
